# Oncolytic virotherapy targeting the IL15 pathway

**DOI:** 10.1016/j.omto.2023.100732

**Published:** 2023-10-11

**Authors:** So Young Yoo, Jeong Heo

**Affiliations:** 1 BIO-IT Foundry Technology Institute, Pusan National University, Busan, Republic of Korea; 2 Department of Internal Medicine, College of Medicine, Pusan National University and Biomedical Research Institute, Pusan National University Hospital, Busan, Republic of Korea

Oncolytic virotherapy (OVT) is a promising cancer treatment in which oncolytic viruses (OVs) act as immunostimulatory agents and selectively replicate and lyse tumor cells without harming normal tissues.^1^ OVs not only directly kill cancer cells but also boost anticancer systemic immunity. Genetic engineering of OVs can enhance their efficacy by including transgenes encoding cytokines, such as granulocyte-macrophage colony-stimulating factor (GM-CSF), interferons (IFNs), tumor necrosis factor (TNF), and interleukins (ILs). Among these, IL-15 is a pleiotropic cytokine that plays an essential role in developing, activating, and promoting survival of T cells, natural killer (NK) cells, and NK-T cells.^2^ For stabilizing and enhancing the bioactivity of IL-15, IL-15 receptor subunit α (IL-15 Rα) can bind to IL-15 independently, form the IL-15/IL-15 Rα complex on the surface of activated monocytes, promote T cell survival, and mediate its signaling pathways.^3^

Shakiba et al. developed recombinant variants of the oncolytic vaccinia virus LIVP strain expressing IL-15 or IL-15 Rα to stimulate IL-15-dependent immune cells.^4^ They evaluated their oncolytic activity either alone or in combination with each other *in vitro* and *in vivo* using the murine CT26 colon carcinoma and 4T1 breast carcinoma. LIVP (vaccinia virus Lister strain from the Institute of Virus Preparation, Moscow, Russia) is an attenuated variant with excellent oncolytic properties. They engineered LIVP-IL-15-RFP and LIVP-IL-15Rα-RFP with deletion of thymidine kinase (TK) for tumor selectivity and inclusion of red fluorescent protein (RFP) as the reporter. They hypothesized that IL-15/IL-15Rα complexes when viruses were used together would produce a synergistic antitumor effect compared with either virus alone.

Results *in vitro* following infection of cell lines showed that arming LIVP with IL-15-RFP increased cytotoxicity, but there was no synergy with the addition of LIVP-IL-15Rα-RFP. In contrast, combination treatment with both viruses was synergistic *in vivo*. Tumor regression was doubled compared to each monotherapy alone, consistent with the increased efficacy being mediated by an immune response. Most tumor-infiltrating T cells in all treated mice were CD4+ cells, whereas CD8+ cells were also significantly elevated in the combination treatment group. IFN-α and IFN-γ, GM-CSF, TNF-α, CXCL9, and CXCL10 were elevated in sera of mice in the combination group. Histologic analyses revealed extensive fields of necrosis with infiltrated immune cells in OV-treated groups but not in the control group. This conversion of a “cold” tumor microenvironment to one that is “hot” provides mechanistic rationale for combining OVs with the expression of various effectors and especially the IL-15/IL-15Rα complex.

Some colorectal cancers with specific genetic mutations, such as microsatellite instability-high (MSI-H) or mismatch repair deficiency (dMMR), have been shown to be more likely to respond to immunotherapy.^5^ The encouraging results from this study suggest that other cancers less prone to response to conventional immunotherapies, such as colon cancer with low MSI, may be amenable to IL-15 combination virotherapy. Triple-negative breast cancer (TNBC) that lack estrogen, progesterone, and HER2 receptors may be another example of a tumor type that could benefit from this novel combination.^6^^,^^7^

## References

[1] Ylösmäki E., Cerullo V. (2020). Design and application of oncolytic viruses for cancer immunotherapy. Curr. Opin. Biotechnol..

[2] Rhode P.R., Egan J.O., Xu W., Hong H., Webb G.M., Chen X., Liu B., Zhu X., Wen J., You L. (2016). Comparison of the Superagonist Complex, ALT-803, to IL15 as Cancer Immunotherapeutics in Animal Models. Cancer Immunol. Res..

[3] Dubois S., Mariner J., Waldmann T.A., Tagaya Y. (2002). IL-15Ralpha recycles and presents IL-15 In trans to neighboring cells. Immunity.

[4] Shakiba Y., Vorobyev P.O., Yusubalieva G.M., Kochetkov D.V., Zajtseva K.V., Valikhov M.P., Kalsin V.A., Zabozlaev F.G., Semkina A.S., Troitskiy A.V. (2023). Oncolytic therapy with recombinant vaccinia viruses targeting the interleukin-15 pathway elicits a synergistic response. Mol. Ther. Oncolytics.

[5] André T., Shiu K.-K., Kim T.W., Jensen B.V., Jensen L.H., Punt C., Smith D., Garcia-Carbonero R., Benavides M., Gibbs P. (2020). Pembrolizumab in Microsatellite-Instability–High Advanced Colorectal Cancer. N. Engl. J. Med..

[6] Loi S., Salgado R., Schmid P., Cortes J., Cescon D.W., Winer E.P., Toppmeyer D.L., Rugo H.S., De Laurentiis M., Nanda R. (2023). Association Between Biomarkers and Clinical Outcomes of Pembrolizumab Monotherapy in Patients With Metastatic Triple-Negative Breast Cancer: KEYNOTE-086 Exploratory Analysis. JCO Precis. Oncol..

[7] Emens L.A., Molinero L., Loi S., Rugo H.S., Schneeweiss A., Diéras V., Iwata H., Barrios C.H., Nechaeva M., Nguyen-Duc A. (2021). Atezolizumab and nab-Paclitaxel in Advanced Triple-Negative Breast Cancer: Biomarker Evaluation of the IMpassion130 Study. J. Natl. Cancer Inst..

